# Malformation artério-veineuse du cuir chevelu

**DOI:** 10.11604/pamj.2013.15.93.2954

**Published:** 2013-07-10

**Authors:** Karima Chakir, Hakima Benchikhi

**Affiliations:** 1 Service de Dermatologie et vénérologie CHU Ibn Rochd Casablanca, Maroc

**Keywords:** Tumeur du cuir chevelu, malformation artério-veineuse, choc vagal, Tumor of the scalp, arteriovenous malformation, vagal shock

## Image en médicine

Nous rapportons une patiente âgée de 46 ans qui présentait depuis la naissance une macule angiomateuse au niveau de l'occiput. A l'âge de 30 ans la lésion a augmenté de volume progressivement au cours des grossesses itératives devenant nodulaire battante et saignant au moindre traumatisme avec plusieurs épisodes de surinfection et épisodes hémorragiques ayant causé une fois un état de choc vagal et perte de conscience. L'examen général retrouvait à son admission une patiente en bon état général avec de bonnes constances hémodynamiques. L'examen dermatologique: au niveau occipital une lésion nodulaire de 6/6 cm de consistance élastique surmontée d'une lésion papuleuse angiomateuse; battante à la palpation chaude, une petite lésion kératosique au centre et un trill positif. Le reste de l'examen somatique est sans particularité. Les diagnostics évoqués: malformation vasculaire superficielle: artério-veineuse; artérielle ou veineuse. L'IRM cérébrale a montré un processus lésionnel du cuir chevelu en regard de l'os occipital extra crânien, constitué de structures serpigineuses vides de signal sur toutes les séquences. L'injection de PDC a déterminé un rehaussement discret de certaines structures qui s'étend sur environ 6.5 cm. Les séquences angiographiques mettent en évidence un peloton vasculaire correspondant à la lésion; sans anomalie parenchymateuse intracrânienne ou artérielle ou veineuse intracérébrale. L'artériographie cérébrale a confirmé aussi l'aspect de malformation artério-veineuse complexe occipitale extra crânienne. La décision des chirurgiens vasculaires était l'abstention thérapeutique et la surveillance à cause le risque hémorragique majeur.

**Figure 1:**
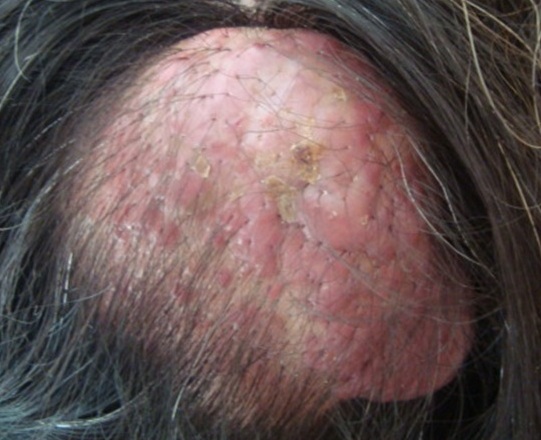
Tumeur du cuir chevelu

